# Intensive care adult patients with severe respiratory failure caused by Influenza A (H1N1)v in Spain

**DOI:** 10.1186/cc8044

**Published:** 2009-09-11

**Authors:** Jordi Rello, Alejandro Rodríguez, Pedro Ibañez, Lorenzo Socias, Javier Cebrian, Asunción Marques, José Guerrero, Sergio Ruiz-Santana, Enrique Marquez, Frutos Del Nogal-Saez, Francisco Alvarez-Lerma, Sergio Martínez, Miquel Ferrer, Manuel Avellanas, Rosa Granada, Enrique Maraví-Poma, Patricia Albert, Rafael Sierra, Loreto Vidaur, Patricia Ortiz, Isidro Prieto del Portillo, Beatriz Galván, Cristóbal León-Gil

**Affiliations:** 1 Critical Care Department, Joan XXIII University Hospital, CIBERes Enfermedades Respiratorias. IISPV. Mallafre Guasch 4 (43007)Tarragona, Spain; 2 Critical Care Department, Son Llatzer Hospital, Crta. Manacor Km 4, (07198) Palma de Mallorca, Spain; 3 La Fe Hospital, CIBERES, Av. Campanar 21 (46009) Valencia, Spain; 4 De la Ribera Hospital. Crta. de Corbera Km 1 (46600) Alzira, Valencia, Spain; 5 Gregorio Marañón Hospital, CIBERES, Calle Doctor Esquerdo 46 (28004) Madrid, Spain; 6 Dr. Negrín Hospital, Barranco de la Ballena s/n (35010) Las Palmas de Gran Canarias, Spain; 7 Infanta Elena, C/Red Corp, J. Andalucía s/n, (21700) Huelva, Spain; 8 Severo Ochoa Hospital, Avd. de Orellana s/n (28911) Leganés, Madrid, Spain; 9 Del Mar Hospital, CIBERES, Passeig Maritim 25-29 (08003) Barcelona, Spain; 10 Insular Hospital de Gran Canarias, Carretera del Sur s/n (35016) Las Palmas de Gran Canarias, Spain; 11 Clinic Hospital, IDIBAPS, CIBERES Enfermedades Respiratorias, C/Villarroel 170 (08036) Barcelona, Spain; 12 San Jorge General Hospital, Av. Martínez de Velazco 36 (22004) Huesca, Spain; 13 Bellvitge University Hospital, CIBERES, Feixa Llarga s/n (08907) Barcelona, Spain; 14 Virgen del Camino Hospital, C/de Irunlarrea 4 (31008) Navarra, Spain; 15 Hospital del Sureste, Ronda del Sur 10 (28500) Arganda del Rey, Madrid, Spain; 16 Puerta del Mar Hospital, Avda Ana de Viya 21 (11009) Cádiz, Spain; 17 Hospital Donostia, Paseo Dr. José Beguiristain s/n (20014) Donostia, San Sebastian, Spain; 18 Josep Trueta University Hospital, Avda. França s/n (17007) Girona, Spain; 19 Ramón y Cajal University Hospital, Ctra. De Colmenar Viejo Km 9,100 (28034) Madrid, Spain; 20 La Paz University Hospital, P de la Castellana 261 (28046) Madrid, Spain; 21 Hospital Nuestra Señora de Valme, Ctra. Cádiz-Bellavist Km 548 (41014) Sevilla, Spain

## Abstract

**Introduction:**

Patients with influenza A (H1N1)v infection have developed rapidly progressive lower respiratory tract disease resulting in respiratory failure. We describe the clinical and epidemiologic characteristics of the first 32 persons reported to be admitted to the intensive care unit (ICU) due to influenza A (H1N1)v infection in Spain.

**Methods:**

We used medical chart reviews to collect data on ICU adult patients reported in a standardized form. Influenza A (H1N1)v infection was confirmed in specimens using real-time reverse transcriptase-polymerase-chain-reaction (RT PCR) assay.

**Results:**

Illness onset of the 32 patients occurred between 23 June and 31 July, 2009. The median age was 36 years (IQR = 31 - 52). Ten (31.2%) were obese, 2 (6.3%) pregnant and 16 (50%) had pre-existing medical complications. Twenty-nine (90.6%) had primary viral pneumonitis, 2 (6.3%) exacerbation of structural respiratory disease and 1 (3.1%) secondary bacterial pneumonia. Twenty-four patients (75.0%) developed multiorgan dysfunction, 7 (21.9%) received renal replacement techniques and 24 (75.0%) required mechanical ventilation. Six patients died within 28 days, with two additional late deaths. Oseltamivir administration delay ranged from 2 to 8 days after illness onset, 31.2% received high-dose (300 mg/day), and treatment duration ranged from 5 to 10 days (mean 8.0 ± 3.3).

**Conclusions:**

Over a 5-week period, influenza A (H1N1)v infection led to ICU admission in 32 adult patients, with frequently observed severe hypoxemia and a relatively high case-fatality rate. Clinicians should be aware of pulmonary complications of influenza A (H1N1)v infection, particularly in pregnant and young obese but previously healthy persons.

## Introduction

As of the 21 August 2009, a total of 177 countries reported 182,166 cases of influenza A (H1N1)v infection, 1799 of which were fatal [1]. Pérez-Padilla and colleagues [2] reported 18 persons with laboratory-confirmed novel influenza A (H1N1) hospitalized at the National Institute of Respiratory Diseases (INER) in Mexico. A Centers for Disease Control and Prevention (CDC) report in May 2009 provided details of the 30 patients who were hospitalized in California, of whom six required admission to an intensive care unit (ICU) and four required mechanical ventilation [3]. In New York City, 909 patients with confirmed pandemic H1N1 influenza have been reported as of 8 July 2009; 225 (25%) have required ICU care and 124 (14%) have required mechanical ventilation with 59 attributed deaths [4].

As of 25 August 2009, 93 deaths linked to the pandemia have been reported in Europe, with 16 deaths in Spain and 59 in the UK [5]. Patients admitted to the ICU are voluntarily reported to a registry of the Spanish Society of Critical Care Medicine (SEMICYUC). This report summarizes the clinical characteristics of a series of the first 32 patients reported to this ICU register, with special interest in those developing severe respiratory failure.

## Materials and methods

Data abstracted for this study were obtained from a voluntary registry instituted by the SEMICYUC after the first known ICU case. Inclusion criteria consisted of: febrile (> 38°C) acute illness; respiratory symptoms consistent with cough, sore throat, myalgia or influenza-like illness; acute respiratory failure requiring ICU admission; plus microbiologic confirmation of novel influenza A (H1N1)v. Data were reported by the attending physician reviewing medical charts, radiologic and laboratory records. The consecutive initial reports notified until 1 August, 2009 were eligible for this study. Children under 15 years old were not enrolled in this registry.

This study was approved by the ethical board of Joan XXIII University Hospital, Tarragona (Spain). Patient identification remained anonymous and informed consent was waived due to the observational nature of the study and the fact that this activity is an emergency public health response. All tests and procedures were ordered by the attending physicians.

Nasopharyngeal-swab specimens were collected at admission and respiratory secretions were also obtained in intubated patients. RT-PCR testing was performed in accordance with published guidelines from the CDC [6]. H1N1 testing was performed in each institution or centralized in a reference laboratory when not available. A 'confirmed case' was defined as an acute respiratory illness with laboratory-confirmed pandemic H1N1 virus infection by real-time RT-PCR or viral culture [7]. Only 'confirmed cases' were included in the current study.

Definition of community-acquired pneumonia was based on current American Thoracic Society and Infectious Disease Society of America guidelines [8].

Primary viral pneumonia was defined in patients presenting during the acute phase of influenza virus illness with acute respiratory distress and unequivocal alveolar opacification involving two or more lobes with negative respiratory and blood bacterial cultures. Secondary bacterial pneumonia was considered in patients with confirmation of influenza virus infection that showed recurrence of fever, increase in cough and production of purulent sputum plus positive bacterial respiratory or blood cultures [9] Bronchoalveolar lavage was not systematically performed because of the high risk of generating aerosols. Respiratory cultures were based on tracheal aspirates obtained immediately after intubation. Acute renal failure was defined as the need for renal replacement therapy following the International Consensus Conference [10].

The ICU admission criteria and treatment decisions for all patients, including determination of the need for intubation and type of antibiotic and antiviral therapy administered, were not standardized and were made by the attending physician.

The following information was recorded: demographic data, comorbidities, time of illness onset and hospital admission, time to first dose of antiviral delivery, microbiologic findings, and chest radiologic findings at ICU admission. Intubation and mechanical ventilation requirements, adverse events during ICU stay (e.g. need for vasopressor drugs, or renal replacement techniques) and laboratory finding at ICU admission were also recorded. To determine the severity of illness, the Acute Physiology And Chronic Health Evaluation (APACHE) II score [11] was determined in all patients within 24 hours of ICU admission. In addition, organ failure was assessed using the Sequential Organ Failure Assessment (SOFA) scoring system [12].

### Statistical analysis

Data analysis was conducted using SPSS 13.0 software (Chicago, IL, USA). Discrete variables are expressed as counts (percentage) and continuous variables as means ± standard deviation (SD) or medians with 25^th ^to 75^th ^interquartile range (IQR). For the demographic and clinical characteristics of the patients, differences between groups were assessed using the chi-squared test and Fisher's exact test for categorical variables and the Student's *t*-test or Mann-Whitney U test for continuous variables. Survival analysis was performed by Kaplan-Meier survival distribution. A *P *value of 0.05 or less was considered to be statistically significant.

## Results

**Figure 1:**
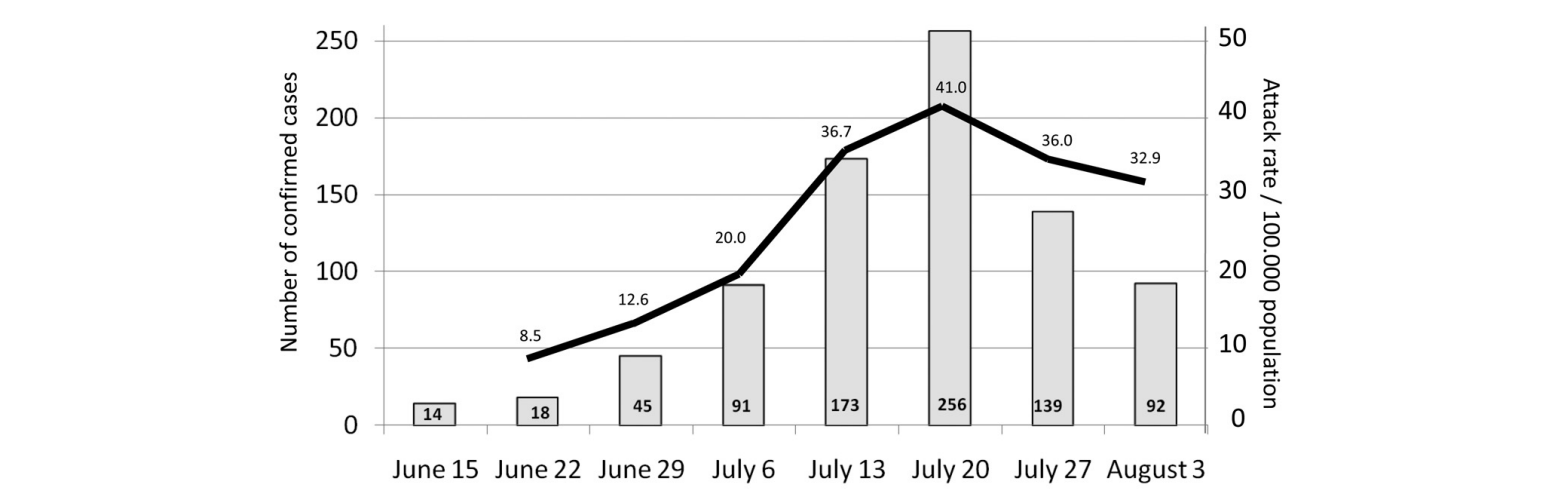
Number of confirmed cases and clinical attack rate in Spain [13].

As of 31 July, 2009, a total of 735 cases of influenza A (H1N1)v were confirmed in Spain. Signs at physician presentation are reported in Table 1. Twelve children (25%) and 36 (75%) older than 14 years required critical care [13] (Figure 1). Data from 32 adults in 20 hospitals were reported to be admitted in ICU with severe respiratory failure and are the focus of this report. All patients were confirmed by real-time PCR for pandemic H1N1 virus. Initial PCR for H1N1 virus at ICU admission was negative in four patients (12.5%). These patients were later confirmed to have the virus through further determination of tracheal secretions. The baseline characteristics of patients are shown in Table 2. The median age was 36 years (IQR = 31 to 52). Sixty patients (50%) were between 18 and 40 years of age, and 22 (68.7%) were less than 52 years of age. Only one patient (3.1%) was older than 65 years. Twenty-one patients (73.3%) were male. Ten patients (31.2%) were reportedly obese (body mass index > 30) and two (6.3%) were pregnant. Asthma (5/32) and exacerbated COPD (4/32) were the main comorbidities reported (Table 3).

**Table 1:** Percentage of signs and symptom of influenza A (H1N1)v in confirmed cases

Fever (96%)
Cough (88%)
Myalgia (69%)
Headache (59%)
Sore throat (58%)
Sudden onset of symptoms (46%)
Malaise (30%)

Source: Ministerio de Salud y Política Social [13].

**Table 2:** Characteristics of 32 patients who had confirmed influenza A (H1N1)v viral primary pneumonitis

Variables	Value
**Age, years**	
Mean (SD)	40 (13.9)
Median (IQR)	36 (31 to 52)
	
**Male sex**, n (%)	21 (73.3)
	
**APACHE II score**, mean (SD)	13.8 (6.4)
	
**SOFA score**, mean (SD)	7.1 (3.3)
	
**Days from onset symptoms to ICU admission**	
Mean (SD)	3.9 (2.2)
Median (IQR)	3 (2 to 6)
**Days from ICU admission to diagnosis**	
Mean (SD)	3.3 (3.1)
Median (IQR)	2 (1 to 6)
**Days from onset symptoms to first antiviral dose**	
Mean (SD)	5.7 (5.1)
Median (IQR)	4 (1 to 8)
**Laboratory finding**, median (IQR)	
Leukocyte count (per mm^3^)	5650 (3000 to 9200)
Platelets count (per mm^3^)	152.000 (124.00 to 227.000)
Serum lactate dehydrogenase (U/L)	953 (728 to 1230)
Serum creatine kinase (U/L)	392 (226 to 3047)
Serum creatinine (mg/dL)	0.87 (0.63 to 1.22)
AST (U/L)	62 (38 to 119)
ALT (U/L)	51 (35 to 111)
**Mechanical ventilation on admission**, n (%)	
NO	8 (25)
Non-invasive	2 (6.2)
Invasive	22 (68.8)
**Adverse event**, n (%)	
Vasopressor drugs	20 (62.5)
Hemodialysis	2 (6.2)
Hemofiltration	5 (15.6)
Refractory hypoxemia requiring prone ventilation	8 (25.0)
Secondary superinfection	3 (9.3)
**Opacity in initial x-ray chest**, n (%)	
1/4 quadrants	5 (15.6)
2/4 quadrants	4 (12.5)
3/4 quadrants	8 (25.0)
4/4 quadrants	15 (46.9)

ALT = alanine aminotransferase; APACHE II = Acute Physiology And Chronic Health Evaluation II; AST = aspartate aminotransferase; ICU = intensive care unit; IQR = interquartile range; NO = nitric oxide; SD = standard deviation; SOFA = Sequential Organ Failure Assessment scoring system.

**Table 3:** Most common risk factors for pandemic influenza A (H1N1)v in the ICU

Risk factor	Cases (n = 32)
Obesity	10
BMI>40	4
BMI 30 to 40	6
Asthma	5
COPD	4
Pregnancy	2
Heart failure	1
Arterial hypertension	1
Chronic renal failure	1
Diabetes mellitus	1
HIV	1
Neuromuscular disease	1
Hematologic disease	1
None	15

BMI = body mass index; COPD = chronic obstructive pulmonary disease; HIV = positive human immunodeficiency virus.

Twenty-nine patients (90.6%) had viral pneumonitis, two patients had exacerbated chronic obstructive pulmonary disease (COPD) and one patient with *Streptococcus pneumoniae *co-infection was reported (documented by respiratory sample culture). All patients received initial empiric antibiotic therapy. Most frequent regimens were beta-lactam plus fluoroquinolones (n = 20, 62.5%); beta-lactam plus macrolides (n = 6; 18.7%) and beta-lactam plus linezolid (n = 5, 15.6%). One patient (3.1%) received levofloxacin as monotherapy. In addition, 11 patients (34.4%) received intravenous steroids at ICU admission. Secondary superinfection with *Pseudomonas aeruginosa *were documented in three patients (9.3%) and one presented with invasive candidiasis. Mean delay between the onset of symptoms and hospital admission was 3.7 ± 2.2 days (IQR = 2 to 5) and between hospital and ICU admission was 1.5 ± 0.8 days (IQR = 1 to 2).

The mean APACHE II score was 13.8 ± 6.4 and the mean SOFA score was 7.1 ± 3.3. Twenty-four patients (75%) developed multiple organ dysfunction syndrome. Twenty patients (62.5%) required vasopressor drugs, and seven patients (21.9%) received renal replacement techniques due to acute renal failure.

**Figure 2:**
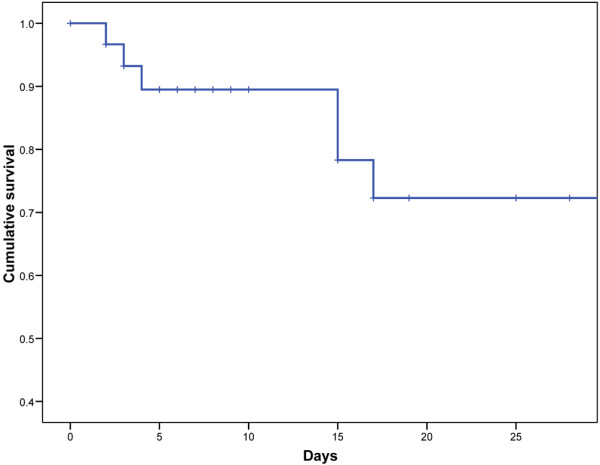
Cumulative survival of 32 intensive care unit patients with influenza A (H1N1)v pneumonia (Censored at 28 days).

Twenty-four patients (75.0%) required mechanical ventilation, and eight (33%) of them required prone position. Extracorporeal membrane oxygenation was not implemented. The characteristics of patients according to ventilatory support required are shown in Table 4. Eight patients (33.3%) received non-invasive mechanical ventilation at ICU admission. Six of these patients (75%) required further orotracheal intubation and invasive mechanical ventilation and two (33%) died. The SOFA score at admission in patients with non-invasive mechanical ventilation failure (8.1 ± 2.3) was significantly higher (*P *= 0.01) than in patients with successful non-invasive mechanical ventilation (2.5 ± 0.7; Table 4). In survivors, the length of mechanical ventilation ranged from 1 to 50 days (median 10, IQR = 1 to 21). As of 27 August, 2009, five patients still remained on mechanical ventilation and eight died due to viral pneumonia (Figure 2). Median age of deceased patients was 35 years after a median of 9.5 days of mechanical ventilation (IQR = 3.2 to 15.7).

**Table 4:** Characteristics of 32 patients according to ventilator support required

Variable	Non-ventilated (n = 8)	Non-invasive ventilation		Initially intubated (n = 16)
			
		Successful (n = 2)	Failure (n = 6)	
APACHE II score				
Mean (SD)	9.5 (4.9) 4 to 16	9.5 (0.7) 9 to 10	15.3 (5.6) 10 to 24	15.2 (7.6) 8 to 38
IQR 25th to 75th				
SOFA score				
Mean (SD)	4.7 (1.7)	2.5 (0.7)*	8.1 (2.3)**	7.8 (3.5)
IQR 25th to 75th	3 to 7	2 to 3	5 to 11	4 to 16
Age, years				
Mean (SD)	39.2 (14.7)	42.5 (13.4)	44.0 (15.1)	38.7 (14.0)
IQR 25th to 75th	17 to 58	33 to 52	10 to 57	16 to 70
Opacity lung quadrants				
Mean (SD)	2.9 (1.2)	2.5 (2.1)	2.8 (0.9)	3.3 (1.1)
LDH, U/L				
Mean (SD)	751 (361)	1140 (374)	918 (408)	2170 (3400)
IQR 25th to 75th	195 to 1166	880 to 1400	354 to 1450	440 to 12200
CK, U/L				
Mean (SD)	2480 (4500)	2800 (3200)	4850 (4200)	2300 (3800)
IQR 25th to 75th	66 to 9300	500 to 5100	122 to 9400	207 to 10800
28-day mortality, n (%)***	0	0	2 (33)	4 (25)

* vs. ** *P *= 0.01

*** Two additional patients died by refractory hypoxemia after 31 and 65 days of mechanical ventilation.

APACHE II = Acute Physiology And Chronic Health Evaluation II; CK = creatine kinase; IQR = interquartile range; LDH = lactate dehydrogenase; SD = standard deviation; SOFA = Sequential Organ Failure Assessment.

**Figure 3:**
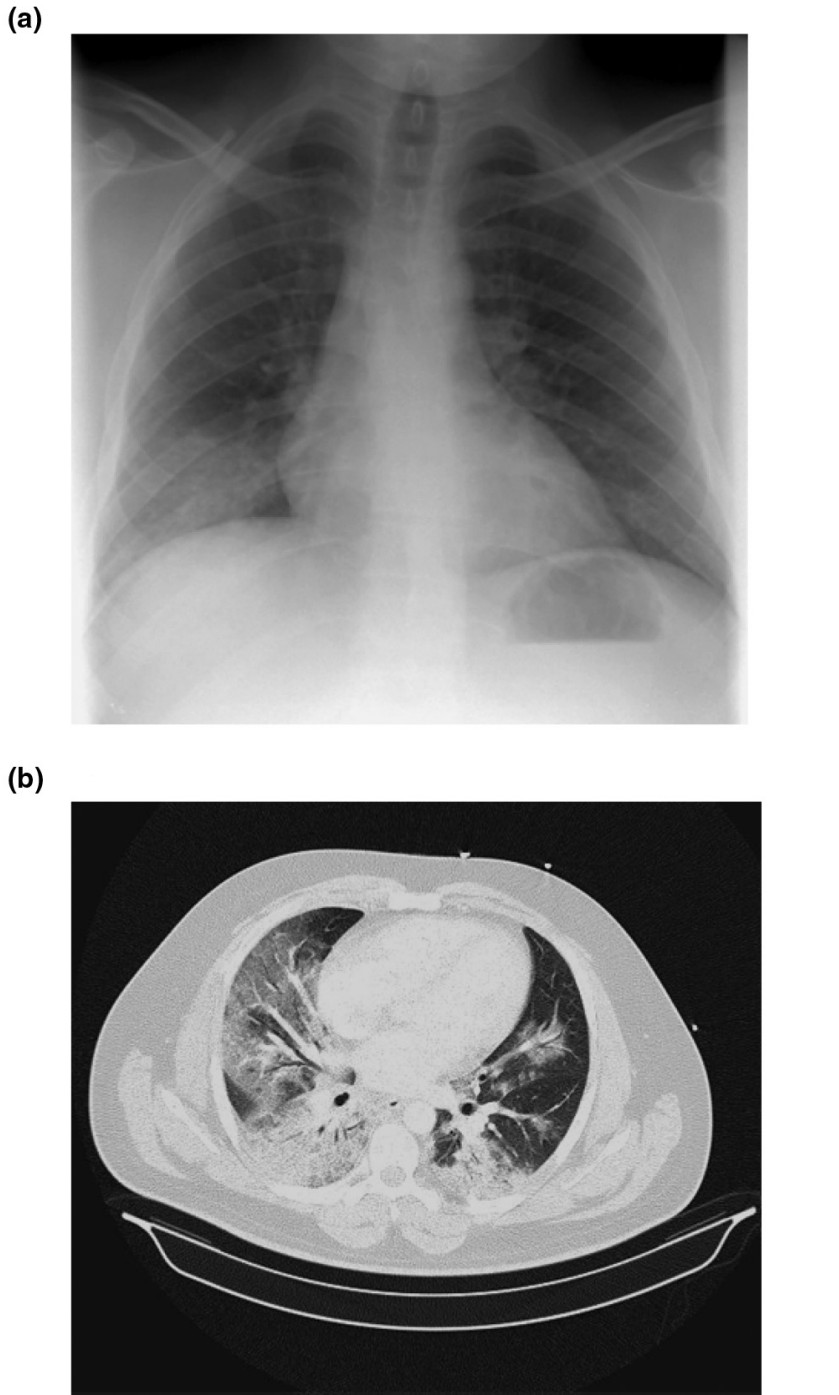
Radiological findings in patients with a viral (H1N1)v pneumonitis. **(a) **Radiograph and **(b) **computed tomography scan of the lung in a patient with viral pneumonitis.

Chest radiograph findings were abnormal in all patients. Patients with viral primary pneumonia had bilateral patchy alveolar opacities (predominantly basal), affecting three or four quadrants in 23 patients (71.8%; Figures 3a). Chest computed tomography scan was performed in only three patients (9.4%) and showed airspace consolidation and ground-glass opacity in a multilobar and bilateral distribution (Figure 3b). Evidence of pulmonary embolism was confirmed in one patient. At the time of ICU admission, 30 patients (93.7%) had elevated lactate dehydrogenase levels (mean 1521.5 ± 2471.9 U/L), 17 (53.1%) above 1000 IU/L. Twenty-three patients (71.8%) had elevated aminotransferases levels (aspartate aminotransferase = 203.5 ± 498.4 U/L and alanine aminotransferase = 156.1 ± 336.2 U/L) and twenty-six patients (81.2%) had increased (mean 2100 ± 34311 U/L) creatinine kinase levels (range 226 to 3047 U/L). C-reactive protein was assessed in 12 patients (37.5%) with a mean of 33.8 ± 25.1 mg/dL and procalcitonin in 8 (25%) with a mean of 1.5 ± 2.1 ng/ml. Nine patients (28.1%) had leucopenia less than 3000 leukocytes/mm^3 ^(mean 7038.4 ± 5847.9 leukocytes/mm^3^), only four patients (12.5%) had more than 10.000 leukocytes/mm^3 ^and four patients (12.5%) had thromobocytopenia less than 100.000 cells/mm^3 ^(mean 175.000 ± 68.000 cells/mm^3^). Eleven patients (32.3%) had elevated creatinine levels(> 1.3 mg/dL) at hospital admission.

In all hospitals, infection control measures were put in place, such as isolation of infected patients, use of personal protective equipment for health care workers, and strict hand hygiene. However, only two infected health care workers were reported.

The estimated median number of days from illness onset to initiation of antiviral treatment was four days (IQR = 2 to 8). Twenty-one patients (65.6%) received antiviral empiric treatment before testing results were available. All patients were administered oseltamivir, including higher-dose oseltamivir (up to 150 mg orally twice a day) in 10 patients (31.2%) with dose adjustment for decreased renal function. The duration of treatment with oseltamivir was 8.0 ± 3.3 days (IQR = 5 to 10).

## Discussion

This report provides details of the first 32 documented patients with influenza A (H1N1)v infection hospitalized in an ICU in Spain. Overall, 1 out of 20 confirmed cases of influenza A (H1N1)v in Spain required critical care. Most of them had refractory hypoxemia and required advanced mechanical ventilation and at least one-third of intubated patients died. This patient group represents the most severely ill subset of persons with influenza A (H1N1)v infection and it is notable for the predominance of males and the high prevalence of obesity.

The pulmonary compromise in this report suggests that severe pulmonary damage occurred as a result of primary viral pneumonia. Although data are not available, this damage also might be attributable to secondary host immune responses (eg, through cytokine dysregulation triggered by high viral replication).

Only nine of the patients in this series had underlying conditions associated with a higher risk for seasonal influenced complications. Obesity was associated with a higher prevalence of comorbid conditions. However, our series confirms that obesity, even in absence of other comorbidities, is frequently associated with viral primary pneumonia in young healthy people. Conditions associated with an increased risk for complications from seasonal influenza include extremes of age, pregnancy, chronic underlying medical conditions and being a resident in a nursing home [4,7]. However, fatal disease associated with influenza A (H1N1)v infection has occurred among previously healthy young people [2].

Further analysis of cases of severe influenza A (H1N1)v infection worldwide is needed. ICU patients in Mexico [2] mainly presented with viral primary pneumonia and mortality was extremely high. Preliminary information from Australia [13] and the USA [3,4] documented other presentations that were also common (acute exacerbation in COPD patients or bacterial co-infection), which were associated with better outcomes. Computed tomography of the lungs very often confirmed pulmonary emboli at admission or as a cause of further deterioration in the USA [3], although it was uncommon in Australia and in our European series. A former report [14] of two patients with rapidly progressive hypoxemia associated with influenza a (H3N2) virus infection noted that they received an initial diagnosis of acute pulmonary embolism.

A common report is of a prolonged time to negative virus excretion [15] associated with the need for higher oseltamivir dosing and longer duration of treatment than standard therapy (75 mg orally twice a day). Indeed, limited data for seasonal influenza suggest that doubling the oseltamivir dose is well tolerated with a comparable adverse event profile. Moreover, some reports [4,16] suggested that doubling the dose may be more effective for H5N1 (avian influence) patients with severe pulmonary disease. Until additional data are available, higher oseltamivir dosage (eg, 150 mg orally twice a day for adults) and extending the duration of therapy should be considered for critically ill patients with influenza A (H1N1)v infection.

Clinicians caring for patients with suspected influenza A (H1N1)v infection should monitor them for rapid respiratory clinical deterioration, especially to increased oxygenation requirements. Empiric antiviral treatment is recommended for all hospitalized patients at admission with suspected influenza A virus infection, including all persons who have received a diagnosis of community-acquired pneumonia, even before diagnostic testing results are available. In hospitalized persons with seasonal or avian influenza A (H5N1), a reduction of mortality has been reported even when oseltamivir treatment was initiated later than 48 hours after illness onset [15-18]. Clinicians should be aware of the false-negative results [3] of rapid influenza diagnostic tests (particularly enzyme immunoassay tests). At least 10% of patients with positive real-time PCR tests in respiratory secretions at intubation have reported prior false-negative tests in nasopharyngeal swabs. Finally, empiric antibiotic agents also should be used for suspected bacterial co-infection.

## Conclusions

Primary viral pneumonia is the main cause of ICU admission in (H1N1)v infected patients, developing severe respiratory failure, which is associated with a relatively high case-fatality. An international registry of patients with influenza A (H1N1)v infection requiring ICU admission is needed. Clinicians should be aware of pulmonary complications of influenza A (H1N1)v infection, particularly in pregnant and young obese but previously healthy persons.

## Key messages

• Patients with pneumonia and high clinical suspicion for influenza A (H1N1)v infection should receive continuous oxygen monitoring and addition of oseltamivir treatment should be not delayed.

• Negative result of RT-PCR at admission should not exclude influenza A (H1N1)v diagnosis due to the presence of false-negative results in 10% of nasopharyngeal-swab specimens.

• Most patients are admitted to the ICU by primary viral pneumonia after a mean of 1.5 days of hospital admission.

• An international registry of ICU patients with influenza A (H1N1)v infection is warranted.

## Abbreviations

APACHE: Acute Physiology And Chronic Health Evaluation; CDC: Centers for Disease Control and Prevention; COPD: chronic obstructive pulmonary disease; ICU: intensive care unit; IQR: interquartile range; RT-PCR: real-time polymerase chain reaction; SEMICYUC: Spanish Society of Critical Care Medicine; SD: standard deviation; SOFA: Sequential Organ Failure Assessment scoring system.

## Competing interests

The authors declare that they have no competing interests.

## Authors' contributions

AR made a substantial contribution to database development, design and analysis, interpretation of data, and wrote the final manuscript. EGM made an important contribution to acquisition and analysis of data. IP, SL, CJ, MA, GJ, RSS, ME, DNSF, MS, FM, AM, GR, AP, SR, VL, OP and GB made an important contribution to acquisition and analysis of data. ALF and MP were involved in revising it critically for important intellectual content. JR and CL made a substantial contribution to the conception, design, analysis and interpretation of data, and revised the final manuscript version. They all approved the final version to be published.

## Authors' information

**H1N1 SEMICYUC working group: **T. Lisboa (Joan XXIII Univsersity Hospital, Tarragona); D. de Mendoza (Joan XXIII University Hospital, Tarragona); M. Borges-Sa (Son Llatzer Hospital, Palma de Mallorca); A. Socias (Son Llatzer Hospital, Palma de Mallorca); A. Torres (Clinic Hospital, Barcelona); J. Ballus Noguera (Bellvitge University Hospital, Barcelona); M. Blasco Navalpotro (Severo Ochoa Hospital, Madrid); I. Jiménez Urra (Virgen del Camino Hospital, Navarra); L. Macaya Redín (Virgen del Camino Hospital, Navarra), A. Tellería (Virgen del Camino Hospital, Navarra); S. Garrido Ramírez de Arellano (San Jorge Hospital, Huesca);M. I. Marquina Lacueva (San Jorge Hospital, Huesca); R. Zaragoza (Dr. Peset Hospital, Valencia); A. Liétor (Ramón y Cajal Hospital, Madrid); R. Ramos (Ramón y Cajal Hospital, Madrid); J. Fugueira(La Paz Hospital, Madrid); M. Cruz Soriano (La Paz Hospital, Madrid); S. Sánchez-Morcillo (De la Ribera Hospital, Valencia); S. Tormo (De la Ribera Hospital, Valencia); P. Marco (Donostia Hospital, San Sebastián); N. González (Donostia Hospital, San Sebastián); J. Garnacho-Montero (Virgen del Rocío Hospital, Sevilla); L. Álvarez-Rocha (Complejo Hospitalario Juan Canalejos, A Coruña); A. Bonet (Josep Trueta Hospital, Girona); JM. Sirvent (Josep Trueta Hospital, Girona); N. López de Arbina (Josep Trueta Hospital, Girona); F. Barcenilla (Arnau de Vilanova Hospital, Lleida); M. Badia (Arnau de Vilanova Hospital, Lleida); F. Mariscal (Infanta Sofía Hospital, Madrid); C. Vaquero (Infanta Sofía Hospital, Madrid); S. García (Infanta Sofía Hospital, Madrid); M. Rodríguez (Infanta Leonor Hospital, Madrid); J. Nolla (Del Mar Hospital, Barcelona); S. Hernández (Del Mar Hospital, Barcelona); J. Vallés (Parc Taulí Hospital, Sabadell); M. Ortíz Hernández (Parc Taulí Hospital, Sabadell); C. Guía (Parc Taulí Hospital, Sabadell); J. Pomarés (S. Cecilio University Hospital, Granada); B. Santamaria (Basurto Hospital, Bilbao); A. Loza (Valme Hospital, Sevilla); P. Galdós (Puerta de Hierro Hospital, Madrid); D. Hernández (La Palma General hospital, La Palma); P. Cobo Castellano (Punta de Europa Hospital, Algeciras); L. Lorente Ramos (Canarias Universitary Hospital, Tenerife); J. Bonastre (La Fe University Hospital, Valencia); M. Palamo (La Fe University Hospital, Valencia); F. Fernández (Delfos Medical Center, Barcelona); J. Ramón (Delfos Medical Center, Barcelona); M. Ortiz Piquer (Xeral Calde Hospital, Lugo); J. Blanco Peréz (Xeral Calde Hospital, Lugo); X. Balanzo (Mataró University Hospital, Barcelona); J. Almirall (Mataró University Hospital, Barcelona); M. Martín Velasco (La Candelaria University Hospital, Tenerife); R. Jordá Marcos (Clinica Rotger, Mallorca); S. Sanchez Alonso (Fuenlabrada Hospital, Madrid); J.J. Cáceres (Insular Hospital, Las Palmas Gran Canaria); C. Castillo Arenal (Txacorritxu Hospital, Vitoria); N. Carrasco (De la Princesa Hospital, Madrid); C. Pascual González (Asturias Central Hospital, Oviedo); J. Nava (Mutua de Terrassa Hospital, Terrassa); J. González de Molina (Mutua de Terrassa Hospital, Terrassa); A. Díaz Lamas (Arquitecto Marcide Hospital, Ferrol); P. Martínez López (Virgen de la Victoria Hospital, Málaga); A. Rovira (l'Hospitalet General Hospital, l'Hospitalet); R. Guerrero (Reina Sofia Hospital, Córdoba); J. Insausti (Navarra Hospital, Navarra); F. García López (Albacete General Hospital, Albacete); J.J. Díaz (Negrín Hospital, Las Palmas Gran Canaria); J. Paez (Dos de Mayo Hospital, Barcelona); P. Ugarte (Valdecillas Hospital, Santander).
